# Draft Genome Sequences of Seven Strains of Dickeya dadantii, a Quick Decline-Causing Pathogen in Fruit Trees, Isolated in Japan

**DOI:** 10.1128/MRA.00609-20

**Published:** 2020-07-09

**Authors:** Takashi Fujikawa, Hiroe Hatomi, Nobuyoshi Ota

**Affiliations:** a Institute of Fruit Tree and Tea Science, National Agriculture and Food Research Organization, Tsukuba, Ibaraki, Japan; University of Maryland School of Medicine

## Abstract

The plant-pathogenic bacterium Dickeya dadantii causes quick decline in fruit trees (e.g., apple, Japanese pear, and peach). In this study, we report on the draft genome sequences of seven strains of D. dadantii that were isolated from fruit trees with typical quick decline symptoms in Japan.

## ANNOUNCEMENT

Dickeya dadantii is a plant-pathogenic bacterium that causes soft-rot disease in various plants (1). In addition to being a soft-rot pathogen, it causes quick decline (QD) in fruit trees, such as apple, peach, and Japanese pear trees (2, 3). The symptoms of QD include red-brown sap leakage from the trunk and/or branches, softening bark, defoliation, leaf and shoot necrosis, and dieback. When QD pathogens have been identified by biochemical and molecular tests, to date only D. dadantii has been isolated (2, 3). However, the life cycle of D. dadantii is not yet well understood. The genetic characteristics of this bacterium will need to be defined to develop effective measures for the control of QD. To this end, we performed whole-genome sequencing (WGS) of seven D. dadantii strains isolated from fruit trees with symptoms of QD.

The strains sequenced in this study were obtained from the Institute of Fruit Tree and Tea Science (National Agriculture and Food Research Organization) (Table 1); they had been isolated from apple, peach, and Japanese pear trees and identified as D. dadantii according to our previous studies (2, 3). The strains were cultivated in YP broth (2) at 27°C for 1 day with agitation at 140 rpm. Then, 1-ml aliquots of each culture were used for DNA extraction with a DNeasy minikit (Qiagen, Hilden, Germany). The genome sequences were determined using WGS. Briefly, the genomic DNA was sequenced using an Ion PGM sequencer with an Ion PGM Hi-Q View OT2 kit, an Ion PGM Hi-Q View sequencing kit, and an Ion 318 Chip kit v2 (Thermo Fisher Scientific, Waltham, MA, USA), according to the manufacturer’s instructions. The sequence reads were quality controlled (quality score, <20) and the adapter sequences were removed using the CLC Genomics Workbench v10 (except for v12 for Kunimi-3 and v20 for BI1-1) (Qiagen). Using the resulting reads, contigs (filtered with a size of >500 bp) were assembled *de novo* using the CLC Genomics Workbench v10 (v12 for Kunimi-3 and v20 for BI1-1) with the default parameters (mapping mode, create simple contig sequences [fast]; automatic bubble size, yes; minimum contig length, 500; automatic word size, yes; performing scaffolding, yes; autodetect paired distances, yes). The draft genomes were annotated using the NCBI Prokaryotic Genome Annotation Pipeline (PGAP) v4. 3 (for strain Kunimi-3) or v4. 11 (for the other strains) (4).

**TABLE 1:** Genome data and accession numbers for seven Dickeya dadantii strains

Strain	Strain information		Genome information					PGAP annotation results					Read information			
	Isolation host	Isolation area	GenBank accession no.	Genome size (bp)	G+C content (mol%)	No. of contigs	*N*_50_ (bp)	Total no. of genes	No. of 5S rRNAs	No. of 16S mRNAs	No. of 23S rRNAs	No. of tRNAs	SRA accession no.	No. of reads	Avg read length (bp)	Genome coverage (×)
BI1-1	Apple	Iwate, Japan	JABEOZ000000000	4,663,807	56.2	271	39,637	4,311	1	2	1	46	SRR11696188	673,373	229.2	33.1
BI3-1	Apple	Iwate, Japan	PHRA00000000	4,771,676	56.3	92	151,183	4,331	4	1	1	61	SRR11692751	5,046,907	298.9	316.1
Aka1-1	Peach	Fukushima, Japan	JABEPA000000000	4,714,768	56.4	268	43,532	4,429	5	1	1	57	SRR11696187	592,688	258.8	32.5
Yana2-2	Peach	Fukushima, Japan	JABEPB000000000	4,825,596	56.4	204	61,681	4,432	3	2	1	48	SRR11696186	1,510,292	255.4	79.9
Kunimi-3	Peach	Fukushima, Japan	SMHE00000000	4,869,298	56.5	141	96,135	4,446	2	1	2	62	SRR11730645	1,941,545	287.6	114.7
Kousui1-1	Japanese pear	Saga, Japan	JABEPC000000000	4,881,710	56.1	193	68,290	4,488	5	1	3	53	SRR11696185	1,308,248	263.2	70.5
Housui2-1	Japanese pear	Saga, Japan	JABEPD000000000	4,882,493	56.2	197	56,134	4,481	4	1	6	49	SRR11696184	1,746,612	258.4	92.4

The WGS analysis indicated that the genome sizes of the seven strains were 4.7 to 4.9 Mbp, with G+C contents of 56.1 to 56.4% (Table 1). The genome size for this species was previously reported in the NCBI database as 4.7 to 5.0 Mbp, with G+C contents of 56.3 to 56.5% (e.g., strains 3937 and DSM 18020 [GenBank accession numbers CP002038 and CP023467, respectively]), which supports the results of our WGS analysis. Moreover, PGAP identified 4,311 to 4,488 genes and multiple rRNA and tRNA genes in these genomes (Table 1). This information can be used to compare the genomes and gene expression patterns of different strains or species. Therefore, the results of our WGS analysis may help to elucidate the characteristics of D. dadantii, a bacterium related to the virulence of QD.

### Data availability.

All WGS projects in this study have been deposited in GenBank. The corresponding read data are available in the Sequence Read Archive (SRA) with the accession numbers provided in Table 1.

## References

[1] Reverchon S, Nasser W 2013 *Dickeya* ecology, environment sensing and regulation of virulence programme. Environ Microbiol Rep 5:622–636. doi:10.1111/1758-2229.12073. 24115612

[2] Ota N, Fujikawa T 2020 Reproduction of bacterial quick decline of fruit trees after soil inoculation with *Dickeya dadantii*. J Gen Plant Pathol 86:199–204. doi:10.1007/s10327-020-00911-9.

[3] Fujikawa T, Ota N, Sasaki M, Nakamura T, Iwanami T 2019 Emergence of apple bacterial quick decline caused by *Dickeya dadantii* in Japan. J Gen Plant Pathol 85:314–319. doi:10.1007/s10327-019-00852-y.

[4] Tatusova T, DiCuccio M, Badretdin A, Chetvernin V, Nawrocki EP, Zaslavsky L, Lomsadze A, Pruitt KD, Borodovsky M, Ostell J 2016 NCBI Prokaryotic Genome Annotation Pipeline. Nucleic Acids Res 44:6614–6624. doi:10.1093/nar/gkw569. 27342282 PMC5001611

